# Spinal radiographic progression in axial spondyloarthritis and the impact of classification as nonradiographic versus radiographic disease: Data from the Swiss Clinical Quality Management cohort

**DOI:** 10.1371/journal.pone.0230268

**Published:** 2020-03-20

**Authors:** Monika Hebeisen, Raphael Micheroli, Almut Scherer, Xenofon Baraliakos, Manouk de Hooge, Désirée van der Heijde, Robert Landewé, Kristina Bürki, Michael J. Nissen, Burkhard Möller, Pascal Zufferey, Pascale Exer, Adrian Ciurea

**Affiliations:** 1 Department of Rheumatology, Zurich University Hospital, Zurich, Switzerland; 2 Statistics Group, Swiss Clinical Quality Management Foundation, Zurich, Switzerland; 3 Rheumazentrum Ruhrgebiet Herne, Ruhr-University Bochum, Bochum, Germany; 4 VIB Inflammation Research Center, Ghent University, Ghent, Belgium; 5 Department of Rheumatology, Leiden University Medical Center, Leiden, The Netherlands; 6 Amsterdam Rheumatology & Clinical Immunology Center, Amsterdam, The Netherlands; 7 Zuyderland Medical Center, Heerlen, The Netherlands; 8 Department of Rheumatology, University Hospital, Geneva, Switzerland; 9 Department of Rheumatology, Allergology and Clinical Immunology, Inselspital, Bern, Switzerland; 10 Department of Rheumatology, Centre Hospitalier Universitaire Vaudois, Lausanne, Switzerland; 11 Rheumatology Practice, Basel, Switzerland; Universita Campus Bio-Medico di Roma, ITALY

## Abstract

**Objective:**

To investigate whether spinal radiographic progression relates to structural damage at the sacroiliac level in axial spondyloarthritis (axSpA).

**Methods:**

Patients classified as nonradiographic (nr-) and radiographic (r-) axSpA in the Swiss Clinical Quality Management cohort with radiographs performed every 2 years, scored according to the modified Stoke Ankylosing Spondylitis Spine Score (mSASSS), were included. The relationship between classification status and spinal progression during 2 years was investigated using binomial generalized estimating equations models with adjustment for sex, ankylosing spondylitis disease activity score (ASDAS) and tumour necrosis factor inhibitor treatment. Baseline spinal damage was considered an intermediate variable and included in sensitivity analyses.

**Results:**

In total, 88 nr-axSpA and 418 r-axSpA patients contributed to data for 725 radiographic intervals. R-axSpA patients were more frequently male, had a longer disease duration and higher structural damage at baseline. Mean (SD) mSASSS change over 2 years was 0.16 (0.62) units in nr-axSpA and 0.92 (2.78) units in r-axSpA, p = 0.01. Nr-axSpA was associated with a significantly lower progression in 2 years (defined as an increase in ≥2 mSASSS units) in adjusted analyses (OR 0.33, 95%CI 0.13; 0.83), confirmed with progression defined as the formation of ≥1 syndesmophyte. Mediation analyses revealed that sacroiliitis exerted its effect on spinal progression indirectly by being associated with the appearance of a first syndesmophyte (OR 0.09, 95%CI 0.02; 0.36 for nr-axSpA vs r-axSpA). Baseline syndesmophytes were predictors of further progression.

**Conclusion:**

Spinal structural damage is mainly restricted to patients with r-axSpA, leading to relevant prognostic and therapeutic implications.

## Introduction

Functional limitation of the spine in axial spondyloarthritis (axSpA) is the consequence of both disease activity and accumulated spinal structural damage [1, 2]. The latter is captured best with the modified Stokes Ankylosing Spondylitis Spine Score (mSASSS) [3], a scoring method that has been shown to be sensitive to change, including the early phases of the disease [4]. Prior spinal structural damage seems to be the most important predictor of further damage in established ankylosing spondylitis [5], now called radiographic (r-) axSpA [6], with mean progression rates of 1–2 mSASSS units over 2 years [7–9]. Male sex, increased acute phase reactants, HLA-B27 positivity, as well as smoking predicted spinal progression in some, but not all investigations [9, 10]. Evidence is accumulating from observational studies that tumour necrosis factor inhibitors (TNFi) might retard spinal damage, if treatment is continued for several years [11–14]. Very limited data on spinal radiographic progression exists for the nonradiographic disease form (nr-axSpA) [4, 10], an entity differentiated from r-axSpA by the absence of definite sacroiliac damage on radiographs [15, 16], according to the modified New York criteria (mNYc) [5], but presenting from a clinical point of view, a burden of disease comparable to r-axSpA despite lower spinal structural damage [17–23]. It remains, however, unclear whether radiographic sacroiliitis is by itself associated with progression of spinal structural damage. From a conceptual point of view, a direct association would be present if, for example, a decreased mobility of the sacroiliac joint could affect physical strain on spinal level to be able to have an impact on syndesmophyte formation [24]. An indirect association would occur if—from the perspective of localization—osteoproliferation would primarily affect the sacroiliac joints and would only later affect the spine. In this case, syndesmophytes would predominantly occur in patients already presenting with sacroiliac damage. Alternatively, both direct and indirect pathways might contribute to the association between sacroiliac and spinal structural damage. Finally, spinal osteoproliferation might be completely disconnected from structural damage at the level of the sacroiliac joints. This would occur if the mechanisms of damage were different at the two locations (for example predominantly erosive disease in the sacroiliac joints and exclusively osteoproliferation at the level of the spine). This does not correspond, however, to the current radiographic and histological data available, as erosive damage followed by putatively reparative osteoproliferation is occurring at both locations of the axial skeleton [25, 26].

The major objective of this study was to compare spinal radiographic progression in nr-axSpA versus r-axSpA in a large observational cohort over a period of 2 years and to investigate whether spinal progression directly or indirectly relates to sacroiliac structural damage by means of statistical mediation analyses.

## Methods

### Study population

Patients with a clinical diagnosis of axSpA recruited into the Swiss Clinical Quality Management (SCQM) cohort [19] between January 2005 and August 2016 were included if they fulfilled the Assessment in SpondyloArthritis international Society (ASAS) 2009 classification criteria for axSpA [27], if they had an available pelvis radiograph to allow for classification as nr-axSpA versus r-axSpA [5] and if they also had at least two sets of spinal radiographs, with a required interval between the radiographs of 2 years ± 1 year. Patients could contribute to several intervals. Information on extent and frequency of clinical assessments has been published previously [19]. Ethics approval was received from the Ethics Committee of the Kanton of Zurich (KEK-ZH-Nr. 2014–0439, amendment BASEC-Nr. PB_2017–00215). Written informed consent for data collection was obtained from all patients prior to recruitment into SCQM.

### Scoring of radiographs

Reading of pelvis radiographs for changes in the sacroiliac joints was performed in a blinded manner centrally by 2 members of the SCQM axSpA scientific board according to the mNYc [5]. In case of discrepancies between the two readers with regard to classification status, the respective pelvis radiographs were independently scored by an independent adjudicator (AC). Reading of all spinal radiographs according to the mSASSS [3] was performed by 2 trained readers (XB and MdH) independently [13]. According to this score, the anterior vertebral corners (VCs) of the cervical and lumbar spine are scored in the lateral view for the presence of erosion and/or sclerosis and/or squaring (1 point), syndesmophyte (2 points) and bridging syndesmophyte (3 points) with a total score per patient ranging from 0 to 72. Radiograph sets with a total mSASSS score of ≥71 were excluded, as no further progression of at least 2 units per 2 years would be possible (N = 8). The readers were blinded to all other information (including class membership to nr- or r-axSpA), but knew the chronological sequence of radiographs, as this is more sensitive to change [28]. Only scores of radiographs with ≤3 missing VC per cervical and lumbar segment were used. Individual missing VCs were imputed using an adaptation algorithm: first, a missing value for a VC was replaced with the value of the previous observation. Second, the mean spinal segment’s progression score (either cervical or lumbar) per patient was calculated. This segmental progression score was added to the imputed value. In a case of a score missing in a patient with a score of 0 in the same VC at a subsequent time point, the score of 0 for the previous time point(s) was assumed. If the baseline score of a VC was missing, the same procedure was applied, subtracting the mean segment progression from the score of year 3 for a particular patient. If a value of this VC was also missing at year 2, then the average of the other available VCs from this spinal segment at baseline was used to replace the missing VC(s). An independent adjudicator (AC) scored all of the radiographs from patients with an absolute difference in mSASSS status scores between the primary readers of ≥5 units in at least one radiograph set. Averaged scores per VC were used and, in case of adjudication, the score of the primary reader closest to the adjudicator.

We defined radiographic progression as an increase in mSASSS of ≥2 units over an interval of 2 years [29]. We also assessed the proportion of patients with formation of ≥1 new syndesmophyte over the same period. Syndesmophytes were only counted if both readers agreed upon their presence.

### Statistical analyses

We assessed reliability between the two readers for spinal radiograph scoring by a Bland-Altman plot on 2-year progression intervals of mSASSS. In addition, we calculated the smallest detectable change for 2-year progression scores and the intraclass correlation coefficient on mSASSS change scores (ICC; type 2, k).

**Fig 1:**
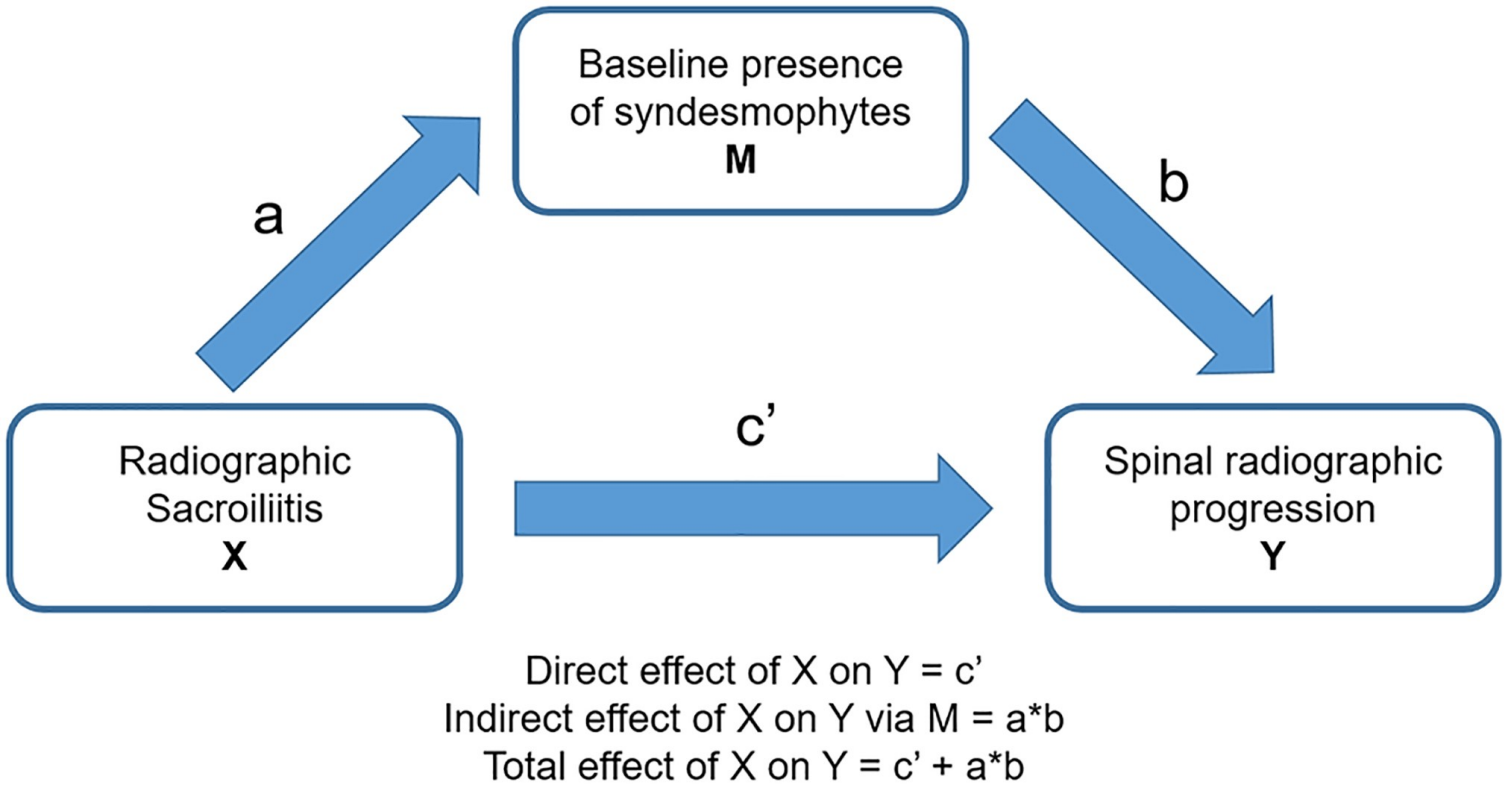
Potential impact of radiographic sacroiliitis on spinal progression. The diagram represents the putative effect of radiographic sacroiliitis (X) on spinal radiographic progression (Y), either directly or indirectly via affecting the baseline presence of syndesmophytes (mediator, M).

The relationship between classification status as nr-axSpA versus r-axSpA and spinal radiographic progression over time was investigated using binomial generalized estimating equations (GEE). These models account for repeated measurements within a patient. An “exchangeable” correlation structure was used, as we assumed that each patient, given all covariates, had an individual constant level of radiographic progression probability for all time points and according to sensitivity analyses of alternative correlation structures in GEE models in our previously published manuscript on radiographic progression in r-axSpA [13]. Progression of ≥2 mSASSS units over 2 years was modelled by using the binomial family and the logistic link function. Based on our previous analysis in r-axSpA [13], the GEE analyses were adjusted for sex, ankylosing spondylitis disease activity score (ASDAS), treatment with TNFi before the respective radiographic interval and the duration of the radiographic interval to account for differences in interval lengths. ASDAS was chosen over BASDAI and CRP as a marker of disease activity in the models for its better association with radiographic progression. Importantly, baseline spinal damage was not included a priori in the main models, as axSpA classification status might have an effect on baseline spinal damage (indirect relational pathway). In this case, baseline spinal damage might act as an intermediate variable and should not be considered in the model to assess the total (direct and indirect) effect of radiographic sacroiliitis on spinal radiographic progression (Fig 1). It was, however, added to the model in sensitivity analyses to investigate a potential direct effect of radiographic sacroiliitis on spinal progression (Fig 1). The indirect association (definite radiographic sacroiliitis present before the appearance of a first syndesmophyte) was tested with the Sobel test with second-order estimator of the standard error, as described by Hayes [30].

Missing baseline covariates were imputed using multiple imputation and models were averaged using Rubin’s rule. Out of 725 intervals, 123 (17%) had a missing ASDAS value. The ASDAS was derived by passive imputation. The same GEE model was also fitted using the subset population with complete data sets to assess the robustness of the results. Additional GEE models were created to also adjust for disease duration, HLA-B27, smoking, physical exercise, treatment with nonsteroidal anti-inflammatory drugs (NSAIDs), BMI, and the presence of peripheral arthritis [13]. R statistical software (R Development Core Team, 2011) was used for all analyses.

## Results

### Baseline characteristics

Inclusion criterial were fulfilled by 506 axSpA patients (418 r-axSpA and 88 nr-axSpA with ≥1 radiographic intervals). Demographic and clinical characteristics of these patients at baseline are presented in Table 1. The prevalence of factors associated with spinal radiographic progression was higher in patients with r-axSpA, such as male predominance, higher baseline radiographic damage and longer disease duration. Moreover, a higher proportion of patients with r-axSpA was already treated with TNFi at baseline. However, disease activity as assessed by the ASDAS was comparable between the two groups.

**Table 1:** Baseline characteristics at first radiograph.

Parameter	N 506	All patients N = 506	nr-axSpA N = 88	r-axSpA N = 418	P
Female sex, %	506	37.4	54.5	33.7	<0.001
Age, years	506	40.2 (11.0)	39.5 (11.1)	40.4 (11.0)	0.52
Symptom duration, years	498	13.3 (10.0)	10.0 (9.9)	14.0 (9.8)	<0.001
HLA-B27 positive, %	452	79.2	71.6	80.7	0.09
BASDAI	427	4.3 (2.2)	4.6 (2.0)	4.2 (2.3)	0.26
ASDAS	408	2.8 (1.0)	2.8 (0.9)	2.8 (1.1)	0.74
CRP (mg/l), median (IQR)	423	8.0 (3.0; 11.0)	5.0 (2.0; 8.0)	8.0 (3.0; 12.0)	0.005
Elevated CRP, %	422	38.9	30.6	40.6	0.14
BASFI	433	3.0 (2.5)	2.8 (2.2)	3.1 (2.5)	0.71
BASMI	435	2.0 (1.9)	1.1 (1.4)	2.2 (2.0)	<0.001
mSASSS median (IQR)	506	0.8 (0.0; 4.0)	0.0 (0.0; 1.0)	1.0 (0.0; 6.0)	<0.001
mean (SD)		5.8 (11.8)	0.9 (1.5)	6.8 (12.7)	
Syndesmophytes present, %	506	30.6	9.1	35.2	<0.001
EQ-5D	429	64.6 (21.0)	61.5 (18.4)	65.2 (21.4)	0.07
Current peripheral arthritis, %	440	30.0	36.8	28.6	0.17
Current enthesitis, %	443	56.4	68.4	54.0	0.02
On csDMARD, %	506	16.0	14.8	16.3	0.87
On NSAIDs, %	401	84.3	80.6	85.1	0.37
On TNFi, %	506	33.4	19.3	36.4	0.002
Current smokers, %	427	37.2	29.7	38.8	0.15
BMI	431	25.2 (4.3)	24.9 (4.5)	25.3 (4.3)	0.48
Number exercise sessions per week, median (IQR)	423	2.0 (0.0; 2.0)	2.0 (0.0; 2.0)	2.0 (0.0; 3.0)	0.08
Patients with different number of radiographic intervals, %	506				0.96
1 interval		69.8	68.2	70.1	
2 intervals		19.8	20.4	19.6	
3 intervals		8.1	9.1	7.9	
4 intervals		2.2	2.3	2.1	
5 intervals		0.2	0.0	0.2	
Length of radiographic interval	506	2.2 (0.5)	2.3 (0.6)	2.2 (0.5)	0.38

Values are the mean (SD), except where indicated otherwise. ASDAS = Ankylosing Spondylitis Disease Activity Score; BASDAI = Bath Ankylosing Spondylitis Disease Activity Index; BASFI = Bath Ankylosing Spondylitis Functional Index; BASMI = Bath Ankylosing Spondylitis Metrology Index; C-reactive protein (CRP) levels; BMI = body mass index; csDMARD = conventional synthetic disease modifying antirheumatic drug; EQ-5D = EuroQol 5-domain; HLA-B27 = human leucocyte antigen B27; MASES = Maastricht Ankylosing Spondylitis Enthesitis Score; modification refers to the inclusion of the plantar fascia in the count; nr-axSpA = nonradiographic axial spondyloarthritis; NSAIDs = Nonsteroidal anti-inflammatory drugs; r-axSpA = radiographic axial spondyloarthritis; TNFi = Tumour necrosis factor inhibitor.

Baseline characteristics of patients with available radiographs were comparable to those of all r-axSpA and nr-axSpA patients included in the SCQM cohort (Table 2).

**Table 2:** Baseline characteristics of all patients with r-axSpA and nr-axSpA at recruitment in SCQM.

Parameter	nr-axSpA N = 431	r-axSpA N = 1155	P
Female sex, %	53.8	30.6	<0.001
Age, years	37.1 (10.8)	40.3 (11.3)	<0.001
Symptom duration, years	8.8 (9.4)	15.0 (11.1)	<0.001
HLA-B27 positive, %	73.1	81.5	<0.001
BASDAI	5.0 (2.2)	4.7 (2.2)	0.14
ASDAS	2.9 (0.9)	3.1 (1.1)	0.02
CRP (mg/l), median (IQR)	4.5 (2.0; 8.0)	8.0 (3.0; 15.0)	<0.001
Elevated CRP, %	27.9	48.1	<0.001
BASFI	3.1 (2.4)	3.6 (2.6)	<0.001
BASMI	1.3 (1.3)	2.5 (2.2)	<0.001
EQ-5D	59.6 (20.9)	60.4 (22.4)	0.49
Current peripheral arthritis, %	38.7	31.6	0.01
Current enthesitis, %	73.5	65.1	0.002
On methotrexate, %	7.9	7.6	0.83
On sulfasalazine, %	5.3	4.8	070
On NSAIDs, %	89.5	88.6	0.64
On TNFi, %	13.9	22.8	0.002
Current smokers, %	31.1	40.6	0.001
BMI	24.7 (4.2)	25.3 (4.5)	0.01
Number exercise sessions per week, median (IQR)	2.0 (0.0; 4.0)	2.0 (0.0; 4.0)	0.92

Values are the mean (SD), except where indicated otherwise. ASDAS = Ankylosing Spondylitis Disease Activity Score; BASDAI = Bath Ankylosing Spondylitis Disease Activity Index; BASFI = Bath Ankylosing Spondylitis Functional Index; BASMI = Bath Ankylosing Spondylitis Metrology Index; C-reactive protein (CRP) levels; EQ-5D = EuroQol 5-domain; HLA-B27 = human leucocyte antigen B27; MASES = Maastricht Ankylosing Spondylitis Enthesitis Score; modification refers to the inclusion of the plantar fascia in the count; nr-axSpA = nonradiographic axial spondyloarthritis; NSAIDs = Nonsteroidal anti-inflammatory drugs; r-axSpA = radiographic axial spondyloarthritis; TNFi = Tumour necrosis factor inhibitor.

### Changes in mSASSS

**Fig 2:**
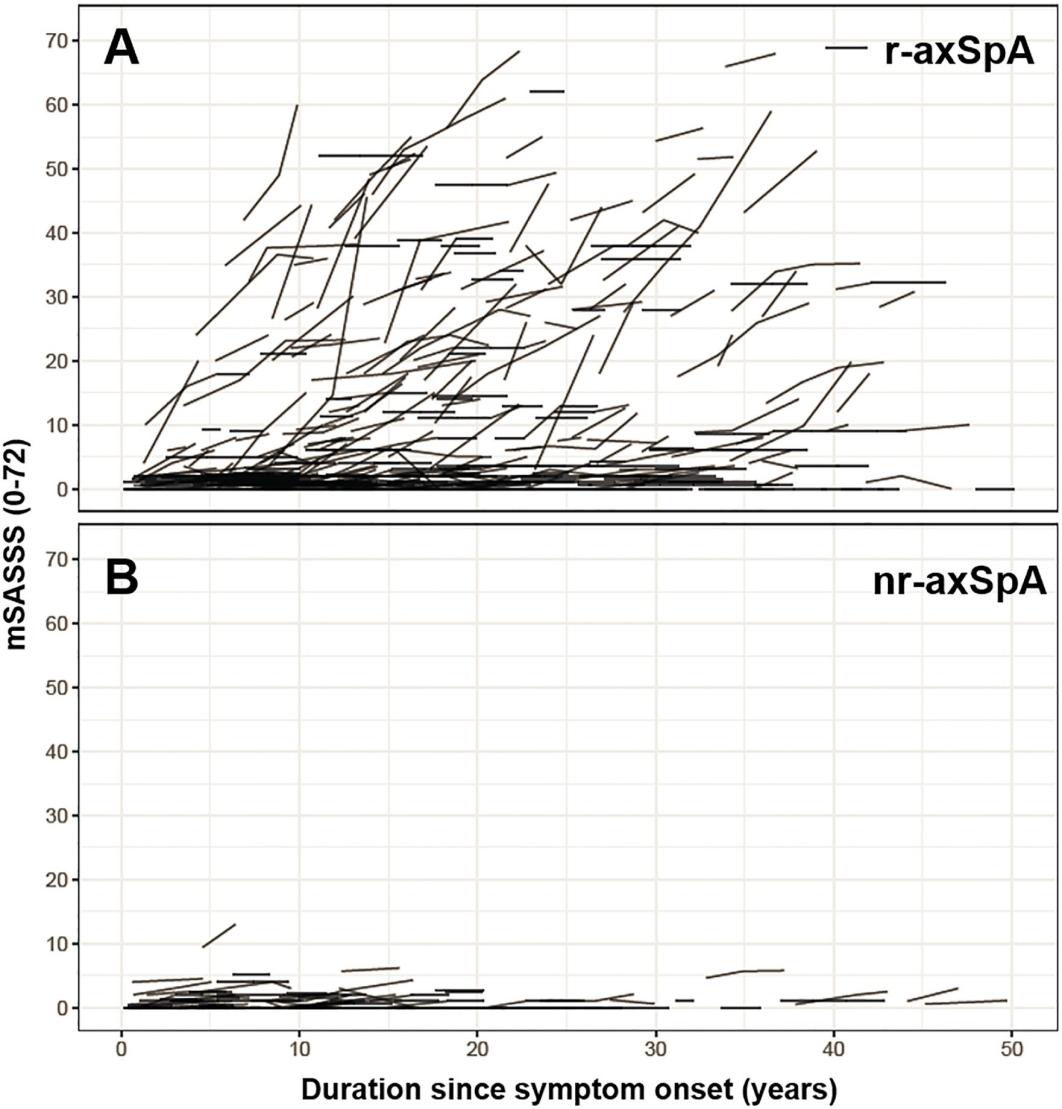
Modified Stoke Ankylosing Spondylitis Spine Score (mSASSS) for individual patients plotted as a function of duration since symptom onset. **A.** Patients with r-axSpA. **B.** Patients with nr-axSpA.

**Fig 3:**
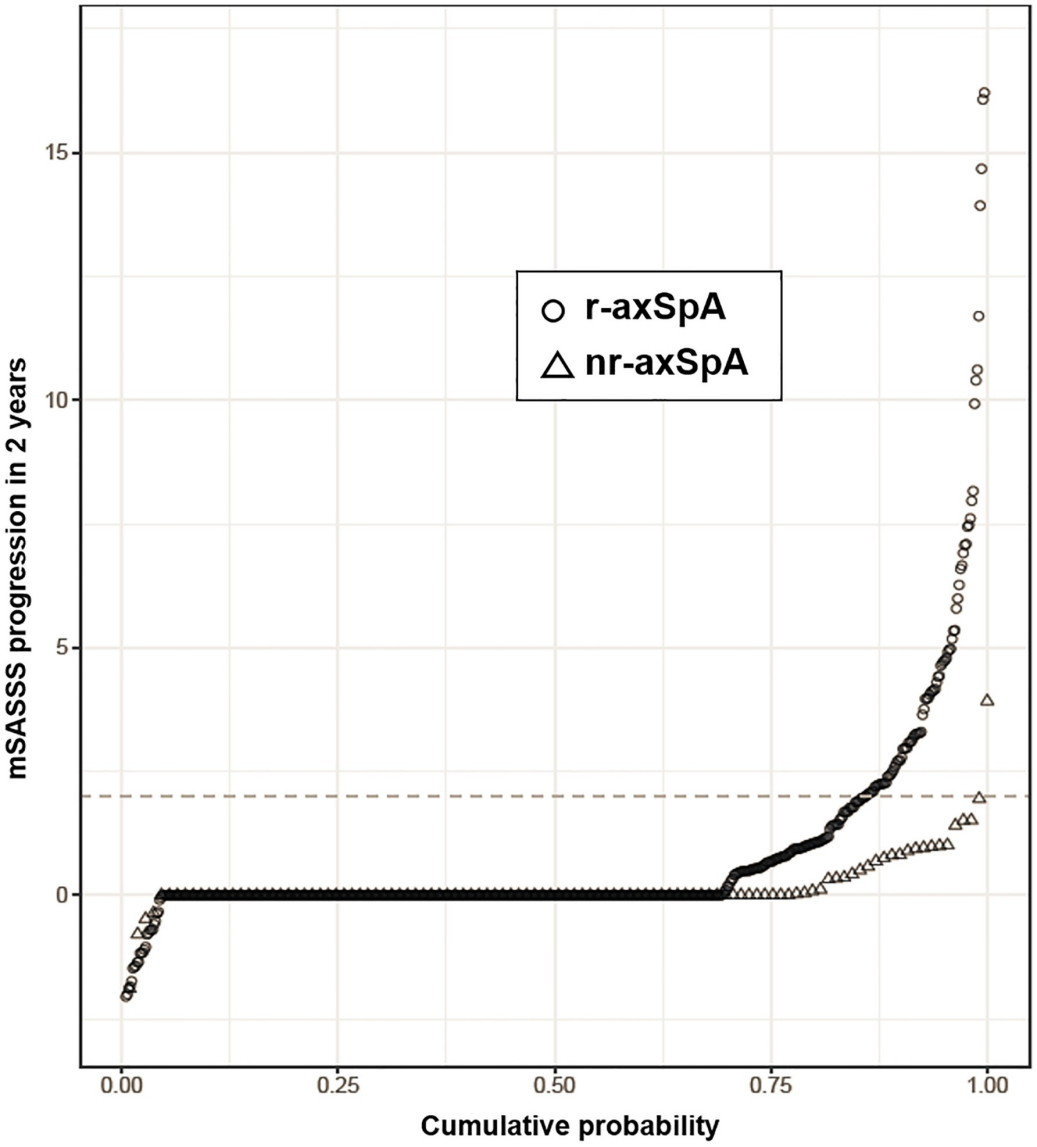
2-year progression in the modified Stoke Ankylosing Spondylitis Spine Score (mSASSS) depicted in a cumulative probability plot. The change in mSASSS values from start to end of individual 2-years radiographic intervals is shown for r-axSpA patients (circles) and for nr-axSpA patients (triangles).

Interobserver agreement with regard to change in mSASSS was good (ICC 0.85). The smallest detectable change of radiographic progression during a X-ray interval was 1.85 mSASSS units. Around 70% of patients in both groups presented with only one radiographic 2-year interval, 20% with 2 intervals and 10% with 3–4 intervals (Table 1). The unadjusted mean (SD) change in the mSASSS over a period of 2 years was significantly lower in patients with nr-axSpA than in patients with r-axSpA (0.16 (0.62) versus 0.92 (2.78) mSASSS units, respectively, p = 0.01). This difference in unadjusted radiographic progression between nr-axSpA and r-axSpA is shown at the patient level in relation to time since symptom onset in Fig 2. The data is also depicted at the level of radiographic intervals in cumulative probability plots in Fig 3.

We next evaluated progression in early versus late disease, defined by a cut-off of 5 years of symptom duration. Mean (SD) spinal progression over 2 years in patients with r-axSpA was lower in early disease than in late disease (0.29 (1.0) units (71 patients (17%), 86 intervals) versus 1.07 (3.0) units (361 patients, 519 intervals) respectively, p = 0.02. In contrast, mean (SD) 2-year progression in nr-axSpA was similar in patients with short and longer symptom duration (0.24 (0.74) units (37 patients (42%), 43 intervals) and 0.10 (0.52) units (52 patients, 66 radiographic intervals), respectively, p = 0.89. A trend for lower radiographic progression was observed in patients with nr-axSpA already on TNFi at the start of the radiographic interval (mean progression of 0.02 (0.55) mSASSS units) in comparison to TNFi-naive nr-axSpA patients (0.21 (0.64) mSASSS units; p = 0.18).

Adjusted longitudinal analyses were performed in 725 radiographic intervals from 506 patients to estimate the joint (direct and/or indirect) effect of sacroiliac damage on spinal progression. After adjustment for the observed differences in prognostic factors for radiographic progression in nr-axSpA and r-axSpA, a significantly lower odds for mSASSS progression was found for nr-axSpA versus r-axSpA (OR 0.33, 95% CI 0.13; 0.83) (Table 3A). The robustness of the analysis was confirmed in a complete case analysis (Table 4). Comparable results were obtained after additional adjustment for disease duration, HLA-B27, smoking status, BMI, physical exercise, presence of peripheral arthritis, treatment with NSAIDs (OR 0.10, 95% CI 0.01; 0.70; Table 5). The direct effect of sacroiliac damage on spinal radiographic damage, assessed by introducing baseline mSASSS in the main model, was, however, not significant (Table 3C). Previous use of TNFi was associated with reduced radiographic progression (OR 0.55, 95% CI 0.34; 0.90), if ASDAS as an intermediate variable for this analysis was removed from the latter model, confirming results found in our previous investigation of the isolated r-axSpA subgroup [13].

**Table 3:** Multivariable analysis for the identification of factors associated with spinal radiographic progression in axSpA.

	Progression defined as ≥2 mSASSS units in 2 years		Progression defined as ≥1 new syndesmophyte in 2 years	
Variable	OR	95% CI	OR	95% CI
	**A**		**B**	
Classification as nr-axSpA vs. r-axSpA (total effect)	0.33	0.13; 0.83	0.45	0.22; 0.93
ASDAS	1.35	1.09; 1.69	1.24	0.99; 1.57
Male sex	3.25	1.76; 6.00	2.65	1.51; 4.64
TNFi use prior to radiographic interval	0.92	0.57; 1.48	0.70	0.44; 1.11
Length of radiographic interval	1.73	0.90; 3.33	1.66	0.93; 2.99
	**C**		**D**	
Classification as nr-axSpA vs. r-axSpA (direct effect)	0.40	0.14; 1.09	0.71	0.31; 1.62
BL mSASSS at start of each radiographic interval	1.07	1.05; 1.09	-	-
BL syndesmophytes at start of each radiogr. interval	-	-	9.77	5.62; 17.0
ASDAS	1.35	1.04; 1.74	1.23	0.97; 1.57
Male sex	1.89	1.07; 3.36	1.38	0.73; 2.61
TNFi use prior to radiographic interval	0.70	0.42; 1.17	0.57	0.35; 0.92
Length of radiographic interval	1.83	0.96; 3.49	2.00	1.05; 3.80

Results from different multivariable models with spinal radiographic progression defined as an increase of ≥2 mSASSS units per 2 years (**A, C**) and progression defined as the formation of ≥1 syndesmophyte in 2 years (**B, D**). Analyses performed in 725 radiographic intervals from 506 patients, according to Fig 1, either assessing the total or the direct effect of classification criteria on spinal progression by ignoring or considering baseline spinal damage (**A,B** or **C,D**, respectively). ASDAS = Ankylosing Spondylitis Disease Activity Score; CI = confidence interval; mSASSS = modified Stoke Ankylosing Spondylitis Spine Score; nr-axSpA = nonradiographic axial spondyloarthritis; OR = odds ratio; r-axSpA = radiographic axial spondyloarthritis; TNFi = Tumour necrosis factor inhibitor.

**Table 4:** Multivariable analysis for the identification of factors associated with spinal radiographic progression in axSpA (complete case analysis).

	Progression defined as ≥2 mSASSS units in 2 years		Progression defined as ≥1 new syndesmophyte in 2 years	
Variable	OR	95% CI	OR	95% CI
	**A**		**B**	
Classification as nr-axSpA vs. r-axSpA	0.20	0.06; 0.68	0.31	0.12; 0.80
ASDAS	1.39	1.10; 1.76	1.26	0.99; 1.61
Male sex	3.24	1.60; 6.55	3.00	1.53; 5.87
TNFi use prior to radiographic interval	1.02	0.61; 1.70	0.85	0.51; 1.41
Length of radiographic interval	1.57	0.75; 3.26	1.55	0.80; 3.01

Analysis performed in 602 radiographic intervals from 427 patients. ASDAS = Ankylosing Spondylitis Disease Activity Score; BMI = Body Mass Index; CI = confidence interval; mSASSS = modified Stoke Ankylosing Spondylitis Spine Score; nr-axSpA = nonradiographic axial spondyloarthritis; OR = odds ratio; r-axSpA = radiographic axial spondyloarthritis; TNFi = Tumour necrosis factor inhibitor.

**Table 5:** Multivariable analysis for the identification of factors associated with spinal radiographic progression in axSpA (model including additional variables).

	A. Progression defined as ≥2 mSASSS units in 2 years		B. Progression defined as ≥1 new syndesmophyte in 2 years	
Variable	OR	95% CI	OR	95% CI
Classification as nr-axSpA vs. r-axSpA	0.10	0.01; 0.70	0.21	0.05; 0.87
ASDAS	1.43	1.04; 1.97	1.32	0.96; 1.181
Male sex	5.54	2.22; 13.8	3.76	1.67; 8.47
TNFi use prior to radiographic interval	0.83	0.44; 1.57	0.68	0.36; 1.31
Length of radiographic interval	1.27	0.56; 2.88	1.71	0.74; 3.95
Disease duration (5 years)	1.31	1.13; 1.52	1.24	1.07; 1.43
Current smoking	0.90	0.49; 1.66	0.56	0.30; 1.06
HLA-B27 positivity	0.60	0.27; 1.32	0.81	0.36; 1.82
Number of exercise sessions per week	1.02	0.89; 1.17	0.86	0.74; 1.01
Peripheral arthritis	0.66	0.34; 1.28	0.58	0.30; 1.12
NSAIDs use at start of radiographic interval	0.85	0.40; 1.82	0.93	0.42; 2.06
BMI 25–30 (Ref. BMI<25)	1.49	0.82; 2.70	0.90	0.49; 1.65
BMI>30 (Ref. BMI<25)	1.38	0.60; 3.22	1.16	0.50; 2.73

Analysis performed in 447 radiographic intervals from 332 patients. ASDAS = Ankylosing Spondylitis Disease Activity Score; BMI = Body Mass Index; CI = confidence interval; HLA-B27 = human leucocyte antigen B27; mSASSS = modified Stoke Ankylosing Spondylitis Spine Score; nr-axSpA = nonradiographic axial spondyloarthritis; NSAID = Nonsteroidal anti-inflammatory drug; OR = odds ratio; r-axSpA = radiographic axial spondyloarthritis; TNFi = Tumour necrosis factor inhibitor.

When analysing the nr-axSpA group separately, a comparable point estimate of the effect size of prior use of TNFi on a change in mSASSS ≥2 units was found after adjustment for baseline mSASSS and sex, but, due to the small sample size, the effect did not achieve statistical significance (OR 0.46, 95% CI 0.04; 5.43, p = 0.54). Baseline mSASSS was associated with an enhanced radiographic progression in this model (OR 1.61, 95% CI 1.06; 2.44, p = 0.03).

### Formation of new syndesmophytes

The proportion of patients with at least one syndesmophyte at baseline was higher in patients with r-axSpA than in patients with nr-axSpA (35.2% vs. 9.1%, p<0.001). At least one new syndesmophyte was identified by both readers in 7/109 (6.4%) radiographic intervals in nr-axSpA and in 102/616 (16.6%) intervals in r-axSpA after 2 years. The proportion of patients with ≥2 syndesmophytes per 2-year radiographic interval was 1/109 (0.9%) in nr-axSpA and 44/616 (7.1%) in r-axSpA. In the adjusted analyses, classification status as nr-axSpA was also associated with a lower odds for the formation of at least one new syndesmophyte (total effect: OR 0.45, 95% CI 0.22; 0.93, Table 3B). The direct effect of sacroiliac damage on spinal progression in the mediation analysis–assessed by introducing the baseline presence of syndesmophytes as a variable to the model, was not significant (Table 3D). In contrast, we found an important effect size of its indirect impact, meaning that syndesmophytes usually do not appear until definite sacroiliac damage has occurred (OR 0.09, 95% 0.02; 0.36 for classification status as nr-axSpA vs r-axSpA).

Prior use of TNFi was associated with reduced spinal progression in the subsequent 2-year radiographic interval, when ASDAS was removed from this model (OR 0.49, 95% CI 0.31; 0.79). We then analysed longitudinal spinal radiographic progression in the subgroup of nr-axSpA patients in a model adjusted for the baseline presence of syndesmophytes, sex and ASDAS (89 intervals from 73 patients). A trend for an enhanced formation of syndesmophytes over 2 years was found for both the presence of syndesmophytes at baseline and disease activity as assessed by the ASDAS (OR 7.07; 95% CI 0.64; 78.5, and OR 3.49, 95% CI 0.68; 18.0, respectively).

## Discussion

Our present study confirms that patients with nr-axSpA display at the group level a lower radiographic spinal progression than patients with r-axSpA [4, 10]. It allowed a more detailed analysis of the intricate relationship between radiographic damage of the sacroiliac joints and of the spine. A previous analysis of the GESPIC cohort revealed an association of structural damage in the sacroiliac joints with function and mobility of the spine, independently of disease activity and structural damage in the spine [31]. As physical strain might lead to inflammation and ultimately to osteoproliferation in spondyloarthritis [24], these changes in function and mobility of the spine due to sacroiliac damage might theoretically increase syndesmophyte formation. However, our mediation analyses in this longitudinal study of patients with nr-axSpA versus r-axSpA failed to demonstrate a statistically significant direct association between sacroiliac damage and spinal structural changes, confirming results previously found in GESPIC [10]. We found a relation between sacroiliac damage and the presence of a first syndesmophyte, as spinal radiographic changes at baseline were virtually restricted to patients already presenting with radiographic sacroiliac changes. Given that baseline spinal damage represents a major predictor of further damage [9, 32], a phenomenon confirmed here, this represents an *indirect* association between sacroiliac and future spinal structural changes. Data from the French DESIR cohort, a prevalence cohort of early axSpA (<3 years) pointed in the same direction. Within the population of patients fulfilling the imaging part of the ASAS classification, spinal progression was lowest in patients lacking definite sacroiliac damage, intermediate in patients fulfilling the modified New York criteria but lacking bone marrow edema on sacroiliac joint MRI and highest in patients having both definite sacroiliitis on X-rays and active sacroiliitis on MRI [4].

Our results highlight the importance of differentiating between r-axSpA and nr-axSpA, which is contrary to the current trend in clinical practice to lump these two entities together. Several issues might have accelerated this trend. Sacroiliac joint scoring according to the modified New York classification criteria by local rheumatologists or radiologists in comparison to central scoring efforts have not always been reliable, particularly with regard to grade 1 versus 2 sacroiliitis [33]. Moreover, from a clinical point of view, disease burden seems similar in nr-axSpA compared to r-axSpA and both disease states are seen as part of a continuum, although this has remained controversial since the publication of the ASAS classification criteria [34–36]. However, recent latent class analyses—performed to avoid inappropriate circularity in the diagnosis of axSpA—yielded several latent classes, indicative of subgroups, forming the “Gestalt” of axSpA [37]. This latter study might suggest together with our current findings, that there might be an axSpA subgroup of patients with a putatively genetically encoded tendency towards osteoproliferation which under certain conditions might evolve to r-axSpA and later form syndesmophytes. This would be compatible with the presence within the nr-axSpA population of a “pre-radiographic” subgroup that might be differentiated from the nr-axSpA population that will never evolve to r-axSpA (“never-radiographic” axSpA or “true” nr-axSpA), exemplified here by a relevant proportion of patients with 20–50 years of symptom duration. The different progression rates from nr-axSpA to r-axSpA found in several previous investigations might therefore be explained by differences in the proportion of patients with “pre-radiographic” axSpA in the populations studied [38]. Future analyses, particularly genetic studies, might help solving this conundrum.

Several additional arguments in favour of differentiating between r- and nr-axSpA from a clinical and prognostic point of view cannot be disregarded. Firstly, the radiographic disease state is, in the absence of an elevated C-reactive protein level, an important predictor of response to treatment. Importantly, not all patients that require a biologic disease-modifying anti-rheumatic drug (bDMARD) present with an elevated CRP. Only patients with an elevated CRP or a sacroiliac joint MRI showing active inflammation have been shown to have a better treatment response in comparison to placebo in nr-axSpA, which is mirrored in the current ASAS treatment recommendations for axSpA [39]. Moreover, an increasing amount of data has been published with regard to the fact the sacroiliac joint bone marrow edema of limited extent is quite unspecific and can occur in other conditions, not related to axSpA [40, 41]. Secondly, deceleration of spinal radiographic progression increasingly becomes a treatment target on its own in addition to the amelioration of clinical symptoms in axSpA, since it has consistently been shown, that treatment with TNF inhibitors for at least 2 years can indeed retard further syndesmophyte formation [11–14].

Our current findings indicate that this treatment target might be of different relevance in nr- vs. r-axSpA, given that syndesmophyte formation is mainly restricted to the r-axSpA disease state. Finally, differentiation between nr- and r-axSpA by central scoring of pelvis radiographs proved here to be reliable to efficiently discriminate between patients with or without spinal progression. As a direct consequence of the current results, we have changed our practice of central scoring in SCQM, to immediately provide the treating rheumatologist feedback with regard to classification status, SCQM being a real-life database used by hospital-based as well as office-based rheumatologists primarily intended to allow treat-to-target strategies. It is important to note that the cut-off of structural MRI lesions allowing reliable classification as nr-axSpA vs. r-axSpA as a substitute for radiographs has already been established [42–44]. A central MRI scoring for routine clinical practice might, however, be more difficult to implement.

Overall spinal progression was very limited in our nr-axSpA group of rather long (10 years) mean disease duration (0.16 mSASSS units over 2 years). Spinal progression in patients fulfilling the ASAS classification criteria but lacking definite radiographic sacroiliitis in the DESIR cohort was also very low (0.1 (±0.7) mSASSS units) [4]. A slightly higher spinal progression (0.5 (±1.6) mSASSS units) was found over 2 years in nr-axSpA patients of the German GESPIC cohort, including patients with a symptom duration of <5 years [10]. In contrast to SCQM and DESIR, no part of this GESPIC population had been treated with bDMARDs during the radiographic interval. We found a trend for lower mean spinal progression in patients treated with TNFi compared to TNFi-naïve patients over 2 years (0.02 versus 0.21 mSASSS units, respectively, p = 0.18). No significant impact of treatment with TNFi on spinal radiographic progression was detected in the adjusted analysis of the nr-axSpA group, although the effect size was comparable to the one found for the r-axSpA group. As overall spinal progression is very limited in nr-axSpA, a potential influence of biologic treatment is very difficult to detect. In RAPID-axSpA, a randomized controlled trial of certolizumab pegol in both AS and nr-axSpA, spinal progression over 4 years was similarly minimal in the nr-axSpA group (0.06 mSASSS units versus 0.98 mSASSS units in AS) [45]. In the absence of an untreated control group, interpretation of the latter finding remains challenging.

Our study has some additional limitations, inherent to the observational nature of the investigation. Selection bias cannot be ruled out, as spinal progression could only be analysed in patients with an available radiograph set. However, baseline characteristics of the included patients were comparable to those of the whole cohort. Moreover, we cannot exclude residual confounding in the adjusted analyses. Information on concurrent inflammation on sacroiliac MRI at the beginning of the radiographic interval might have improved the robustness of our results. This information is, however, not available in SCQM, as data on prior sacroiliac inflammation on MRI is collected from the treating rheumatologist for purposes of classification, but the time-point of the examination largely remains unknown. Expecting, based on previous publications [10], a rather limited radiographic progression in nr-axSpA, we chose to score the radiographs in known chronological order. This has been shown to have a higher sensitivity to change [28]. Moreover, scorers were blinded to all clinical data. The advent of spinal progression assessment with low-dose CT opens new research opportunities [46, 47].

## Conclusion

The formation of syndesmophytes in axSpA is mostly restricted to patients with structural damage of the sacroiliac joints, further increasing the prognostic relevance of differentiating between nr-axSpA and r-axSpA.

## Supporting information

S1 File Raw data file. (XLSX)
